# Cognitive and Functional Change Over Time in Cognitively Healthy Individuals According to Alzheimer Disease Biomarker-Defined Subgroups

**DOI:** 10.1212/WNL.0000000000207978

**Published:** 2023-12-22

**Authors:** Mark A. Dubbelman, Heleen M.A. Hendriksen, John E. Harrison, Everard G.B. Vijverberg, Niels D. Prins, Lior A. Kroeze, Lois Ottenhoff, Mardou M.S.S.A. Van Leeuwenstijn, Inge M.W. Verberk, Charlotte E. Teunissen, Elsmarieke M. van de Giessen, Argonde C. Van Harten, Wiesje M. Van Der Flier, Sietske A.M. Sikkes

**Affiliations:** From the Alzheimer Center Amsterdam, Neurology (M.A.D., H.M.A.H., J.E.H., E.G.B.V., L.A.K., L.O., M.M.S.S.A.V.L., I.M.W.V., C.E.T., A.C.V.H., W.M.V.D.F., S.A.M.S.), and Departments of Radiology & Nuclear Medicine (E.M.v.d.G.), Epidemiology & Data Science (W.M.V.D.F.), and Neurochemistry Laboratory, Department of Laboratory Medicine (I.M.W.V., C.E.T.), Amsterdam UMC, Vrije Universiteit Amsterdam; Neurodegeneration, Amsterdam Neuroscience; Brain Research Center (N.D.P., L.O.); and Department of Clinical, Neuro and Developmental Psychology (S.A.M.S.), Faculty of Behavioral and Movement Sciences, Vrije Universiteit, Amsterdam, the Netherlands.

## Abstract

**Background and Objectives:**

It is unclear to what extent cognitive outcome measures are sensitive to capture decline in Alzheimer disease (AD) prevention trials. We aimed to analyze the sensitivity to changes over time of a range of neuropsychological tests in several cognitively unimpaired, biomarker-defined patient groups.

**Methods:**

Cognitively unimpaired individuals from the Amsterdam Dementia Cohort and the SCIENCe project with available AD biomarkers, obtained from CSF, PET scans, and plasma at baseline, were followed over time (4.5 ± 3.1 years, range 0.6–18.9 years). Based on common inclusion criteria for clinical trials, we defined groups (amyloid, phosphorylated tau [p-tau], *APOE* ε4). Linear mixed models, adjusted for age, sex, and education, were used to estimate change over time in neuropsychological tests, a functional outcome, and 2 cognitive composite measures. Standardized regression coefficients of time in years (β_time_) were reported as outcome of interest. We analyzed change over time with full follow-up, as well as with follow-up limited to 1.5 and 3 years.

**Results:**

We included 387 individuals (aged 61.7 ± 8.6 years; 44% female) in the following (partly overlapping) biomarker groups: *APOE* ε4 carriers (n = 212), amyloid-positive individuals (n = 109), amyloid-positive *APOE* ε4 carriers (n = 66), CSF p-tau–positive individuals (n = 127), plasma p-tau–positive individuals (n = 71), and amyloid and CSF p-tau–positive individuals (n = 50), or in a control group (normal biomarkers; n = 65). An executive functioning task showed most decline in all biomarker groups (β_time_ range −0.30 to −0.71), followed by delayed word list recognition (β_time_ range −0.18 to −0.50). Functional decline (β_time_ range −0.17 to −0.63) was observed in all, except the CSF and plasma tau–positive groups. Both composites showed comparable amounts of change (β_time_ range −0.12 to −0.62) in all groups, except plasma p-tau–positive individuals. When limiting original follow-up duration, many effects disappeared or even flipped direction.

**Discussion:**

In conclusion, functional, composite, and neuropsychological outcome measures across all cognitive domains detect changes over time in various biomarker-defined groups, with changes being most evident among individuals with more AD pathology. AD prevention trials should use sufficiently long follow-up duration and/or more sensitive outcome measures to optimally capture subtle cognitive changes over time.

## Introduction

Alzheimer disease (AD) has been biologically defined by the accumulation of amyloid in combination with the aggregation of cortical tau, eventually resulting in cognitive decline and dementia.^1,2^ Several risk factors of amyloid accumulation have been identified, including the ε4 allele of the *APOE* gene.^3^ Secondary prevention and disease-modifying therapies for AD are currently being developed and evaluated in numerous clinical trials.^4^

While biological effects of various treatments on pathologic markers have been demonstrated,^5-9^ evidence of clinical benefit remains limited,^10,11^ with recent exceptions.^12,13^ Treatments' failures to meet their end points have often been ascribed to the treatment mechanism, but other factors such as trial design^14^ and target population heterogeneity^15^ also play a role. Another important issue is the selection of outcome measures: Traditionally used outcome measures may not be able to capture treatment effects, especially in preclinical stages, where the expected clinical changes are subtle.^16,17^ In 2018, the US Food and Drug Administration published guidelines for the development of drugs specifically for the treatment of early AD, which included a call for the development and use of outcome measures that are sensitive enough to measure subtle cognitive and functional changes.^18^

Hence, there is a need for cognitive and functional end points that are sensitive to changes over time in early disease stages.^19-21^ It is conceivable that, depending on participant inclusion criteria (e.g., *APOE*-ε4 carriership, elevated cortical amyloid and/or tau), different cognitive outcomes may be most appropriate because these individuals represent distinct disease subgroups and/or disease risk stages. Furthermore, subtle changes may not be measurable within the currently common clinical trial duration, which varies between 78 and 104 weeks for prodromal and mild AD^10,22-24^ and between 80 and 240 weeks (mean duration of 159 weeks) for preclinical AD.^25^

Therefore, the aim of our study was to investigate how cognitive and functional outcome measures change over time in various biomarker-defined groups, based on the screening or inclusion criteria of secondary prevention trials in preclinical AD. We hypothesized that inclusion criteria may have differential effects on cognitive outcomes, that is, outcome measures may show larger or smaller effects in specific biomarker-defined groups. To assess potential influence of the commonly used follow-up duration in current clinical trials, we performed analyses with follow-up duration limited to 78 and 156 weeks (i.e., 1.5 and 3 years).

## Methods

### Study Participants and Procedures

Data were obtained from the Amsterdam Dementia Cohort (ADC) and the SCIENCe cohort, which is embedded within the ADC. Study procedures for both cohorts are described in detail elsewhere.^26,27^ Briefly, patients who visited the outpatient memory clinic of the Alzheimer Center Amsterdam for dementia screening and gave consent to use their clinical data for research purposes were included in the ADC. All patients underwent a standard diagnostic workup, including medical history, neurologic examination, laboratory screening tests, and neuropsychological assessment. Those who were 45 years and older, had cognitive complaints, but had no neurologic or psychiatric diagnosis were invited to participate in the SCIENCe cohort. In addition to the parent cohort inclusion criteria, selection criteria for this study included (1) the presence of self-perceived difficulties in cognitive functioning, defined as having visited the memory clinic for dementia screening, in the absence of objective cognitive deficits; (2) the availability of at least 1 biomarker for AD at baseline; and (3) having at least 1 follow-up visit with neuropsychological assessment. Based on exclusion criteria frequently used in secondary prevention trials, participants were excluded if their baseline Mini-Mental State Examination was <26 and/or if their baseline Geriatric Depression Scale (GDS) score was ≥6 or if baseline Mini-Mental State Examination (MMSE), GDS, or all biomarker data were missing, resulting in a total of 387 included individuals.

### Standard Protocol Approvals, Registrations, and Patient Consents

The study was approved by the medical ethical review board of the VU University Medical Center. All participants provided written informed consent for the use of their clinical data for research purposes before undergoing any study procedures.

### Defining Biomarker Groups

The presence of abnormal amyloid was determined either from CSF (n = 316) or an amyloid PET scan (n = 134). For CSF, local cutoffs were used to distinguish between normal and abnormal amyloid: <813 pg/mL for the Innotest ELISA assay that was used until June 2018^28^ or <1,092 pg/mL for the Elecsys assay that was used from June 2018 onward.^29^ For amyloid PET, an experienced nuclear radiologist who was blinded to clinical information visually rated scans, acquired using one of [^18^F]flutemetamol, [^18^F]florbetapir, or [^18^F]florbetaben as tracer. When both CSF and PET were available for the same individual and within 1 year of each other, we used the PET results to determine amyloid status.

Phosphorylated tau (p-tau) was measured in CSF (n = 316) and plasma (n = 214). CSF p-tau was categorized as abnormal if >52 pg/mL for the ELISA assay or if >19 pg/mL for the Elecsys assay.^28,29^ EDTA plasma samples were collected through venipuncture. Samples were measured using the Simoa pTau181 V2 Advantage kit on the Simoa HDx analyzer,^30^ in duplicates. Samples were categorized as abnormal if >1.64 pg/mL (unpublished results; based on Youden cut point to define an abnormal amyloid PET or CSF status in a population with subjective cognitive decline [n = 318; n = 64 amyloid-positive] and mild cognitive impairment [n = 282; n = 166 amyloid-positive]). Plasma p-tau was treated as a separate biomarker.

*APOE* (n = 370) genotyping was performed using Sanger sequencing on an ABI130XL after automated genomic DNA isolation, PCR testing, and having been checked for size and quantity using a QlAxcel DNA Fast Analysis kit. Patients with either 1 or 2 ε4 alleles were classified as *APOE* ε4 carriers.

**Figure 1:**
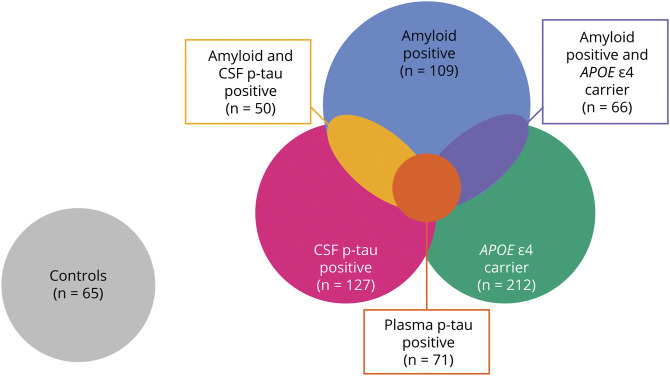
Venn Diagram Showing the Biomarker and Control Groups p-tau = phosphorylated tau.

Based on the specified biomarkers and *APOE* ε4 carriage, we defined the following partly overlapping biomarker groups: (1) *APOE* ε4 carriers (n = 212), (2) amyloid-positive individuals (n = 109), (3) amyloid-positive *APOE* ε4 carriers (n = 66), (4) CSF p-tau–positive individuals (n = 127), (5) plasma p-tau–positive individuals (n = 71), and (6) amyloid and CSF p-tau–positive individuals (n = 50). Participants who did not carry an *APOE* ε4 allele and who were both amyloid and (CSF and plasma) p-tau–negative were included in a control group (n = 65; Figure 1).

### Outcome Measures

Outcome measures were assessed annually. Global cognitive functioning was rated using the MMSE with total scores ranging from 0 to 30, where higher scores represent better cognitive functioning.^e1^ Episodic memory was measured using the immediate and delayed recall and delayed recognition of the Dutch version of the Auditory Verbal Learning Test (AVLT)^e2^ and the immediate and delayed recall of the Rivermead Behavioral Memory Test (RBMT).^e3^ Semantic memory measures included semantic fluency (animal naming)^e4^ and the Boston Naming Test (BNT).^e5^ Executive functioning was measured using part B of the Trail Making Test (TMT),^e6^ card III of the Stroop Color-Word Test (SCWT),^e7^ and phonetic fluency. Attention and working memory tests included the digit span (DS) forward and backward,^e8^ part A of the TMT,^e6^ and cards I and II of the SCWT.^e7^ Test scores were reversed as necessary, so a higher score on all tests represented better performance.

Everyday functioning was measured using the Amsterdam Instrumental Activities of Daily Living Questionnaire (A-IADL-Q), which is a 30-item study partner-reported instrument covering a range of cognitively complex everyday activities.^e9,e10^ Total scores are normally distributed in a memory clinic sample around a mean of 50 with a SD of 10 and range from approximately 20 to 80, with higher scores representing better everyday functioning.

The number of individuals who completed the outcome measures and the total number of observations over the entire study duration per outcome measure are listed in Table 1.

**Table 1:** Number of Individuals and Observations per Outcome Measure

Outcome measure	No. of individuals	No. of observations
MMSE	387	1,406
A-IADL-Q	202	500
LDST	297	446
DS, forward	387	993
TMT-A	386	1,335
SCWT-I	376	1,183
SCWT-II	373	1,174
DS, backward	386	990
TMT-B	385	1,314
SCWT-III	373	1,170
Phonetic fluency	363	1,120
AVLT, immediate recall	387	1,329
AVLT, delayed recall	387	1,319
AVLT, recognition	387	1,323
RBMT, immediate recall	205	663
RBMT, delayed recall	204	663
Semantic fluency	386	1,317
BNT	251	722

Abbreviations: A-IADL-Q = Amsterdam Instrumental Activities of Daily Living Questionnaire; AVLT = Auditory Verbal Learning Test; BNT = Boston Naming Test; DS = digit span; LDST = Letter Digit Substitution Test; MMSE = Mini-Mental State Examination; RBMT = Rivermead Behavioral Memory Test; SCWT = Stroop Color-Word Task; TMT = Trail Making Test.

We also re-created 2 composites using the available tests: the Preclinical Alzheimer's Cognitive Composite (PACC5)^e11^ and the Cognitive-Functional Composite (CFC).^e12,e13^ Our version of the PACC5 included the immediate and delayed recall of the AVLT as a replacement for the Free and Cued Selective Reminding Test and Logical Memory Scale, respectively; the Letter Digit Substitution Test (LDST) as a substitution for the Digit Symbol Substitution Test; the MMSE; and category fluency. Our version of the CFC included the recognition and delayed recall of the AVLT as a replacement for ADAS-Cog word recognition and recall; the LDST as a substitution for the Digit Symbol Substitution Test, phonetic fluency, semantic fluency, and DS backward; as well as the A-IADL-Q total score. The A-IADL-Q was included as available and was not required for calculation of the CFC. It did not include a measure of orientation (as a replacement for the ADAS-Cog orientation). Both composites use *Z*-scores (mean ± SD 0 ± 1) that were calculated using baseline mean and SD in the control group. Higher scores represent better performance.

### Data Analysis

All analyses were performed in R version 4.2.1.^e14^ Baseline differences between each of the biomarker groups and controls were tested using *t* tests or χ^2^ tests, with corrections for multiple testing, as appropriate. To investigate responsiveness to change over time of each outcome measure, a series of linear mixed models (LMMs) were performed with random intercepts. Random slopes were included if there were sufficient observations to model them. All LMMs were performed in each separate biomarker group, with adjustments for sex, years of education, and baseline age. We report standardized coefficients (β) and their 95% CIs to allow for comparison between models; β can range −1 to 1, with larger absolute numbers representing a stronger association.

#### Comparing With Biomarker-Negative Controls

To investigate whether observed changes were disease-specific (and not due to aging), we additionally investigated each biomarker group's change over time in relation to change over time in the control group in separate models. To do so, we ran LMMs with a time-by-group interaction, where the dichotomous group variables included the control group and one of the biomarker groups. The time-by-group interaction was the main outcome of interest and was adjusted for sex, years of education, and baseline age.

#### Mimicking a Trial: Reducing Follow-up Duration

Finally, to assess potential influence of the commonly used follow-up duration in current clinical trials, we performed analyses with follow-up duration limited to 156 and 78 weeks (i.e., 3 and 1.5 years, respectively).

### Data Availability

Anonymized data not published within this article will be made available by request from any qualified investigator.

## Results

We included 387 participants aged 61.7 ± 8.6 years at baseline, and 171 of them (44.2%) were female. Table 2 summarizes the baseline characteristics of the total sample and the (partly overlapping) subgroups. Compared with controls, participants in all biomarker groups were older, and amyloid and CSF p-tau–positive individuals had a lower baseline MMSE score than controls while the other biomarker groups did not differ from controls in baseline MMSE (Table 2). There were no group differences between controls and any of the biomarker groups in sex distribution or educational attainment (Table 2).

**Table 2:** Baseline Characteristics

Characteristic	Overall	Controls	Biomarker groups						
			Any positive biomarker	*APOE* ε4	Amyloid	Amyloid + *APOE* ε4	CSF p-tau	Plasma p-tau	Amyloid + CSF p-tau
N (%)	387 (100.0)	65 (16.8)	322 (83.2)	213 (55.0)	109 (28.2)	66 (17.1)	127 (32.8)	71 (18.3)	50 (12.9)
Age	61.7 ± 8.6	57.5 ± 7.6	62.6 ± 8.5^a^	62.2 ± 8.4^a^	64.8 ± 7.6^a^	65.4 ± 5.5^a^	64.2 ± 7.6^a^	64.4 ± 7.7^a^	66.8 ± 6.3^a^
Female, n (%)	171 (44.2)	24 (36.9)	147 (45.7)	100 (46.9)	56 (51.4)	33 (50.0)	59 (46.5)	35 (49.3)	27 (54.0)
Education, median (IQR)	13 (10–13)	13 (10–17)	13 (10–13)	13 (10–13)	13 (10–17)	13 (10–13)	13 (10–13)	13 (10–13)	13 (10–16)
MMSE	28.6 ± 1.2	28.6 ± 1.2	28.5 ± 1.2	28.5 ± 1.2	28.4 ± 1.1	28.4 ± 1.2	28.4 ± 1.2	28.5 ± 1.2	28.1 ± 1.3^a^
Amyloid, n positive/n total (%)	109/337 (32.3)	0/65 (0.0)	109/272 (40.1)	66/165 (40.0)	109/109 (100.0)	66/66 (100.0)	50/127 (39.4)	28/69 (40.6)	50/50 (100.0)
CSF p-tau, n positive/n total (%)	127/316 (40.2)	0/65 (0.0)	127/251 (50.6)	65/155 (41.9)	50/98 (51.0)	33/62 (53.2)	127/127 (100.0)	33/61 (54.1)	50/50 (100.0)
Plasma p-tau, n positive/n total (%)	71/214 (33.2)	0/65 (0.0)	71/149 (47.7)	36/86 (41.9)	28/45 (62.2)	21/31 (67.7)	33/67 (49.3)	71/71 (100.0)	16/21 (76.2)
*APOE* ε4 carrier, n (%)	212/387 (54.8)	0/65 (0.0)	212/322 (65.8)	213/213 (100.0)	66/109 (60.6)	66/66 (100.0)	64/127 (50.4)	36/71 (50.7)	33/50 (66.0)
Follow-up duration, y, range	4.5 ± 3.1 (0.6–18.9)	4.0 ± 2.4 (0.9–9.2)	4.6 ± 3.2 (0.6–18.9)	4.7 ± 3.2 (0.6–18.2)	4.6 ± 3.2 (0.8–18.9)	4.8 ± 3.0 (0.8–18.3)	5.0 ± 3.4 (0.6–18.9)	3.7 ± 2.1 (0.9–10.3)	5.5 ± 3.9 (1.1–18.9)

Abbreviations: IQR = interquartile range; MMSE = Mini-Mental State Examination; p-tau = phosphorylated tau.

Data shown as mean ± SD, except as noted otherwise. Amyloid was available for n = 337 (87.1%), CSF p-tau for n = 316 (81.7%), plasma p-tau for n = 214 (55.3%), and *APOE* ε4 carriership and sex for n = 387 (100.0%). Biomarker groups are partly overlapping.

a Different from controls, adjusted *p* value <0.05.

**Figure 2:**
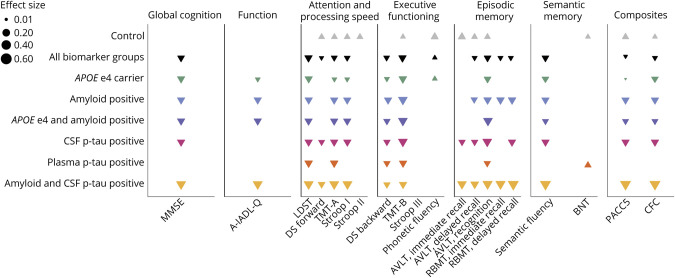
Change Over Time on All Tests, Across All Biomarker Groups Standardized coefficients, adjusted for age, sex, and education, are visualized as triangles. The magnitude of the coefficient is reflected by the triangle's size. Upward-pointing triangles represent an improvement in performance; downward-pointing triangles represent a decrease in performance. When no triangle is displayed, there was no change over time (i.e., the 95% CI included change in both directions). Full data are shown in eTable 1. A-IADL-Q = Amsterdam Instrumental Activities of Daily Living Questionnaire; AVLT = Auditory Verbal Learning Test; BNT = Boston Naming Test; CFC = Cognitive-Functional Composite; DS = digit span; LDST = Letter Digit Substitution Test; MMSE = Mini-Mental State Examination; PACC5 = Preclinical Alzheimer's Cognitive Composite; p-tau = phosphorylated tau; RBMT = Rivermead Behavioral Memory Test.

We investigated all outcome measures in the individual biomarker subgroups. The adjusted standardized coefficients of all tests in the control group and all biomarker groups are presented in Figure 2 and Table 3. Unadjusted and adjusted coefficients are given in eTable 1 (links.lww.com/WNL/D264). TMT-B showed most decline over time across biomarker groups, with the smallest decline occurring in plasma p-tau–positive individuals (β = −0.30, 95% CI −0.46 to −0.14) and the largest in amyloid and CSF p-tau–positive individuals (β = −0.71, 95% CI −1.01 to −0.42). Another executive functioning test, DS backward, also showed consistent decline in all biomarker groups, but effect sizes were somewhat smaller. Regarding the attention and processing speed tests, TMT-A showed a decline in all biomarker groups, with the smallest decline observed in *APOE* ε4 carriers (β = −0.15, 95% CI −0.27 to −0.04) and the largest in amyloid and CSF p-tau–positive individuals (β = −0.50, 95% CI −0.82 to −0.17). Other tests for attention and processing speed showed consistent decline in performance over time, including SCWT card I, with rate of change ranging from −0.15 (in *APOE* ε4 carriers; 95% CI −0.25 to −0.05) to −0.42 (in amyloid and CSF p-tau–positive individuals; 95% CI −0.72 to −0.12), and the LDST, with rate of change ranging from −0.29 (in *APOE* ε4 carriers; 95% CI −0.40 to −0.19) to −0.49 (in amyloid and CSF p-tau–positive individuals; 95% CI −0.68 to −0.30). In the episodic memory domain, the AVLT recognition, and not AVLT immediate or delayed recall, showed most change over time, with change ranging from −0.18 (in plasma p-tau–positive individuals; 95% CI −0.36 to −0.00) to −0.50 (in amyloid and CSF p-tau–positive individuals; 95% CI −0.75 to −0.25). Of the tests for semantic memory, semantic fluency showed a decline in all biomarker groups, except the plasma p-tau–positive group. Rates of change ranged from −0.17 (in *APOE* ε4 carriers; 95% CI −0.24 to −0.11) to −0.49 (in amyloid and CSF p-tau–positive individuals; 95% CI −0.67 to −0.32).

**Table 3:** Adjusted Standardized Coefficients for Change Over Time in Neuropsychological Tests Over the Full Follow-Up Duration, in All Biomarker Groups and Controls

	Controls	All biomarker groups	*APOE* ε4 carriers	Amyloid positive	Amyloid positive and *APOE* ε4 carrier	CSF p-tau positive	Plasma p-tau positive	Amyloid and CSF p-tau positive
Global cognition								
MMSE	0.00 (−0.11 to 0.11)	−0.22 (−0.34 to −0.11)	−0.25 (−0.37 to −0.12)	−0.38 (−0.58 to −0.18)	−0.37 (−0.59 to −0.15)	−0.29 (−0.48 to −0.10)	−0.07 (−0.27 to 0.13)	−0.60 (−0.92 to −0.28)
Function								
A-IADL-Q	−0.07 (−0.23 to 0.08)	−0.10 (−0.21 to 0.02)	−0.17 (−0.31 to −0.03)	−0.31 (−0.51 to −0.12)	−0.32 (−0.58 to −0.06)	−0.13 (−0.33 to 0.07)	−0.04 (−0.21 to 0.14)	−0.63 (−0.86 to −0.39)
Attention and processing speed								
LDST	−0.06 (−0.20 to 0.09)	−0.31 (−0.40 to −0.23)	−0.29 (−0.40 to −0.19)	−0.37 (−0.51 to −0.23)	−0.32 (−0.48 to −0.17)	−0.31 (−0.44 to −0.18)	−0.34 (−0.50 to −0.17)	−0.49 (−0.68 to −0.30)
DS, forward	0.12 (0.02 to 0.21)	−0.07 (−0.13 to −0.01)	−0.04 (−0.10 to 0.03)	−0.09 (−0.20 to 0.01)	−0.01 (−0.11 to 0.08)	−0.15 (−0.23 to −0.07)	−0.13 (−0.27 to −0.01)	−0.24 (−0.36 to −0.11)
TMT, A^a^	0.16 (0.06 to 0.26)	−0.20 (−0.30 to −0.09)	−0.15 (−0.27 to −0.04)	−0.33 (−0.56 to −0.09)	−0.27 (−0.47 to −0.07)	−0.28 (−0.45 to −0.11)	−0.34 (−0.52 to −0.16)	−0.50 (−0.82 to −0.17)
SCWT, I^a^	0.15 (0.03 to 0.27)	−0.14 (−0.23 to −0.05)	−0.15 (−0.25 to −0.05)	−0.25 (−0.42 to −0.09)	−0.22 (−0.39 to −0.04)	−0.24 (−0.41 to −0.07)	−0.16 (−0.33 to 0.01)	−0.42 (−0.72 to −0.12)
SCWT, II^a^	0.14 (0.03 to 0.25)	−0.04 (−0.15 to 0.06)	−0.02 (−0.15 to 0.11)	−0.12 (−0.33 to 0.10)	0.04 (−0.27 to 0.35)	−0.06 (−0.28 to 0.17)	−0.13 (−0.30 to 0.05)	−0.07 (−0.53 to 0.38)
Executive functioning								
DS, backward	−0.01 (−0.11 to 0.09)	−0.14 (−0.19 to −0.08)	−0.11 (−0.17 to −0.05)	−0.23 (−0.32 to −0.14)	−0.18 (−0.29 to −0.08)	−0.18 (−0.27 to −0.10)	−0.20 (−0.33 to −0.07)	−0.28 (−0.41 to −0.15)
TMT, B^a^	0.11 (0.02 to 0.19)	−0.28 (−0.37 to −0.19)	−0.33 (−0.44 to −0.23)	−0.49 (−0.66 to −0.32)	−0.57 (−0.78 to −0.35)	−0.41 (−0.58 to −0.24)	−0.30 (−0.46 to −0.14)	−0.71 (−1.01 to −0.42)
SCWT, III^a^	0.14 (−0.00 to 0.28)	−0.05 (−0.15 to 0.06)	−0.00 (−0.15 to 0.14)	−0.16 (−0.37 to 0.04)	−0.01 (−0.31 to 0.29)	−0.04 (−0.27 to 0.18)	−0.03 (−0.23 to 0.16)	−0.18 (−0.60 to 0.24)
Phonetic fluency	0.24 (0.13 to 0.34)	0.07 (0.01 to 0.13)	0.09 (0.02 to 0.16)	0.00 (−0.11 to 0.11)	0.02 (−0.10 to 0.14)	0.02 (−0.09 to 0.13)	0.04 (−0.09 to 0.16)	−0.09 (−0.34 to 0.17)
Episodic memory								
AVLT, immediate recall	0.28 (0.16 to 0.41)	0.04 (−0.05 to 0.12)	0.03 (−0.07 to 0.12)	−0.12 (−0.28 to 0.05)	−0.15 (−0.35 to 0.05)	−0.15 (−0.30 to −0.00)	0.11 (−0.05 to 0.26)	−0.48 (−0.73 to −0.23)
AVLT, delayed recall	0.19 (0.05 to 0.33)	−0.09 (−0.17 to −0.02)	−0.08 (−0.17 to 0.01)	−0.19 (−0.34 to −0.03)	−0.17 (−0.38 to 0.05)	−0.21 (−0.34 to −0.07)	−0.04 (−0.17 to 0.09)	−0.39 (−0.66 to −0.13)
AVLT, recognition	0.15 (0.01 to 0.29)	−0.25 (−0.33 to −0.16)	−0.26 (−0.35 to −0.16)	−0.34 (−0.48 to −0.19)	−0.39 (−0.53 to −0.25)	−0.34 (−0.49 to −0.19)	−0.18 (−0.36 to −0.00)	−0.50 (−0.75 to −0.25)
RBMT, immediate recall	−0.07 (−0.24 to 0.10)	−0.12 (−0.19 to −0.04)	−0.08 (−0.17 to 0.01)	−0.24 (−0.42 to −0.06)	−0.24 (−0.48 to 0.00)	−0.13 (−0.29 to 0.03)	−0.13 (−0.26 to 0.01)	−0.53 (−0.94 to −0.13)
RBMT, delayed recall	−0.02 (−0.19 to 0.16)	−0.09 (−0.17 to −0.02)	−0.06 (−0.15 to 0.04)	−0.25 (−0.41 to −0.08)	−0.26 (−0.49 to 0.00)	−0.21 (−0.37 to −0.05)	−0.06 (−0.20 to 0.07)	−0.65 (−1.01 to −0.29)
Semantic memory								
Semantic fluency	0.09 (−0.02 to 0.19)	−0.18 (−0.24 to −0.11)	−0.17 (−0.24 to −0.11)	−0.33 (−0.44 to −0.21)	−0.27 (−0.39 to −0.15)	−0.23 (−0.33 to −0.13)	0.02 (−0.14 to 0.18)	−0.49 (−0.67 to −0.32)
BNT	0.10 (0.02 to 0.19)	0.04 (−0.06 to 0.14)	0.01 (−0.06 to 0.09)	0.02 (−0.19 to 0.23)	0.18 (−0.11 to 0.46)	0.01 (−0.10 to 0.13)	0.07 (0.00 to 0.16)	−0.14 (−0.38 to 0.11)
Composites								
PACC5	0.18 (0.08 to 0.27)	−0.11 (−0.20 to −0.02)	−0.12 (−0.21 to −0.02)	−0.29 (−0.47 to −0.11)	−0.26 (−0.45 to −0.06)	−0.26 (−0.42 to −0.10)	0.04 (−0.11 to 0.19)	−0.62 (−0.93 to −0.31)
CFC	0.17 (0.08 to 0.26)	−0.15 (−0.22 to −0.07)	−0.16 (−0.24 to −0.07)	−0.26 (−0.42 to −0.10)	−0.26 (−0.44 to −0.08)	−0.27 (−0.41 to −0.12)	−0.07 (−0.21 to 0.08)	−0.52 (−0.84 to −0.20)

Abbreviations: A-IADL-Q = Amsterdam Instrumental Activities of Daily Living Questionnaire; AVLT = Auditory Verbal Learning Test; BNT = Boston Naming Test; CFC = Cognitive-Functional Composite; DS = digit span; LDST = Letter Digit Substitution Test; MMSE = Mini-Mental State Examination; PACC5 = Preclinical Alzheimer's Cognitive Composite; RBMT = Rivermead Behavioral Memory Test; SCWT = Stroop Color-Word Task; TMT = Trail Making Test.

All coefficients are shown as coefficient (95% CI), adjusted for baseline age, sex, and education.

a Coefficient inversed, so all negative values indicate a decline in performance.

Global cognition, assessed with the MMSE, declined over time in all biomarker groups, except for plasma p-tau–positive individuals, with rates of change ranging from −0.25 in *APOE* ε4 carriers to −0.60 in amyloid and CSF p-tau–positive individuals. Everyday functioning declined over time among *APOE* ε4 carriers, amyloid-positive individuals, and amyloid and CSF p-tau–positive individuals, with rate of change ranging from −0.17 to −0.63. Performance on both composites declined over time in all biomarker groups, except the plasma p-tau–positive group, with the largest rate of change observed in amyloid and CSF p-tau–positive participants (PACC5: β = −0.62, 95% CI −0.93 to −0.31; CFC: β = −0.52, 95% CI −0.84 to −0.20).

A few tests did not show any change over time in the biomarker groups. These included SCWT cards II and III, phonetic fluency, and BNT. These last 2 tests even showed an improvement over the full follow-up duration: phonetic fluency among *APOE* ε4 carriers (β = 0.09, 95% CI 0.02–0.16) and BNT in plasma p-tau–positive individuals (β = 0.07, 95% CI 0.00–0.16).

### Comparing With Biomarker-Negative Controls

When including a time-by-group (control vs each biomarker group) interaction, change on TMT-B and DS backward differed in all biomarker groups from controls. Of tests for attention and processing speed, TMT-A, DS forward, and SCWT card I showed a decline in all biomarker groups, whereas controls did not while LDST showed a decline among amyloid-positive, plasma p-tau–positive, and amyloid and CSF p-tau–positive individuals that was not observed in controls. The rate of change in AVLT recognition differed between all biomarker groups and controls. Semantic fluency declined in all biomarker groups, except the plasma p-tau–positive group, but not in controls.

While change over time on SCWT card III and BNT in the biomarker groups did not differ from change over time on those tests in controls, we did observe a reduced increase in performance on SCWT card II and phonetic fluency, as compared with controls, in most biomarker groups.

The rate of change in everyday functioning only differed between amyloid and CSF p-tau–positive individuals and controls. Performance on both composites differed in change over time compared with controls, except for change over time on the PACC5 in plasma p-tau–positive participants. All coefficients of change in each biomarker group compared with controls are provided in eTable 2 (links.lww.com/WNL/D265).

### Mimicking a Trial: Reducing Follow-Up Duration

**Figure 3:**
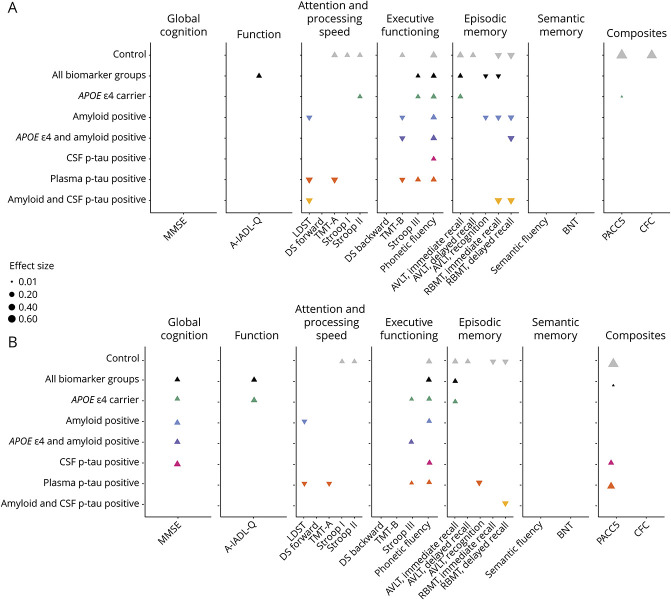
Change Over Time on All Tests, Across All Biomarker Groups, With Follow-Up Duration Limited to 156 Weeks (A) and 78 Weeks (B) Standardized coefficients, adjusted for age, sex, and education, are visualized as triangles. The magnitude of the coefficient is reflected by the triangle's size. Upward-pointing triangles represent an improvement in performance; downward-pointing triangles represent a decrease in performance. When no triangle is displayed, there was no change over time (i.e., the 95% CI included change in both directions). Full data are shown in eTable 1. A-IADL-Q = Amsterdam Instrumental Activities of Daily Living Questionnaire; AVLT = Auditory Verbal Learning Test; BNT = Boston Naming Test; CFC = Cognitive-Functional Composite; DS = digit span; LDST = Letter Digit Substitution Test; MMSE = Mini-Mental State Examination; PACC5 = Preclinical Alzheimer's Cognitive Composite; p-tau = phosphorylated tau; RBMT = Rivermead Behavioral Memory Test.

Figure 3 shows the adjusted standardized coefficients of all tests in all biomarker groups with 156 and 78 weeks of follow-up duration. All raw unadjusted and adjusted coefficients are provided in eTable 1 (links.lww.com/WNL/D264). With follow-up duration limited to 156 weeks (3 years), most effects described above disappeared: Rates of change decreased or even inverted. Notably, phonetic fluency performance improved over 156 weeks across all biomarker groups, except amyloid and CSF p-tau–positive individuals.

We observed similar changes over time when further limiting follow-up duration to 78 weeks (1.5 years). The MMSE showed improved performance in most biomarker groups, but not in controls, plasma p-tau–positive individuals, or amyloid and CSF p-tau–positive individuals.

When including the time-by-group (control vs each biomarker group) interaction, we observed that over 156 weeks, rates of change only differed on a few outcome measures, including TMT-A and B and SCWT card I. Remarkably, over 78 weeks, controls showed improvement over time on AVLT immediate and delayed recall, as well as SCWT cards I and II, whereas performance in all biomarker groups remained stable over this time frame. All coefficients of change in each biomarker group compared with controls over 156 and 78 weeks are provided in eTable 3 (links.lww.com/WNL/D266).

## Discussion

We found a decline in cognitive and functional outcome measures among cognitively normal individuals that differed slightly depending on the presence of specific AD biomarker abnormalities: While outcome measures in all cognitive domains showed changes over time in all groups, individuals with abnormal biomarkers that represent later-stage pathology showed more change on outcome measures than individuals with fewer abnormal biomarkers. Tests for attention and processing speed (LDST, TMT, A, SCWT I), executive functioning (DS, backward, TMT, B), and episodic memory (ALVT, recognition) showed most prominent decline over time in all biomarker groups, and not in the control group. These findings persisted when including a time-by-group interaction, showing that the decline is likely not due to normal aging. When limiting follow-up duration to 78 or even 156 weeks, many changes disappeared, or switched to an improvement over time, illustrating the relevance of long trial durations in preclinical stages of AD.

We hypothesized that inclusion criteria may have differential effects on cognitive outcomes, with outcome measures potentially showing stronger effects in certain biomarker-defined groups. We did not observe such differential effects: In all biomarker-defined groups, outcome measures in all cognitive domains showed some decline over time in the full follow-up. We did find that cognitive and functional changes are most evident among individuals with more abnormal AD biomarkers. *APOE* ε4 carriers and plasma p-tau–positive individuals showed only limited decline, particularly on tests of episodic memory. In amyloid-positive individuals, CSF p-tau–positive individuals, and amyloid and CSF p-tau–positive individuals, an increasing number of episodic memory tests showed significant decline over time. Episodic memory decline is the most salient symptom in early AD, and previous studies also showed increased memory decline with an increased number of biomarkers affected.^31^ The largest changes (of up to two-thirds of a SD per year) were present in amyloid and CSF p-tau–positive individuals, whereas both the CSF p-tau–positive individuals and amyloid-positive individuals showed relatively small changes over time, corresponding to less than one-third of a SD per year. In line with other studies, cognitive changes in cognitively normal individuals were most pronounced in individuals who were p-tau and amyloid-positive.^32^ Composite measures showed similar decline as individual neuropsychological tests. The functional measure declined in all biomarker groups, except for the CSF or plasma p-tau groups, and most notably in amyloid and CSF p-tau–positive individuals. Thus, the differential effects among the biomarker-defined groups presented here may not reflect distinct cognitive profiles, but rather may seem to represent staging of AD pathology consistent with the biological framework proposed by the National Institute on Aging and Alzheimer's Association.^33^ Furthermore, individual neuropsychological tests do not seem to have inferior sensitivity to composite measures and, in earlier stages, might even be more sensitive.

Our study raises the question of whether subsequent cognitive decline over a few years is necessarily present in initially unimpaired individuals who are at risk of or have biomarker evidence of AD. Second, if cognitive decline is indeed present, the question remains whether current practice allows for the successful detection of such decline.

As an answer to the first question, we only observed cognitive decline with a sufficiently long follow-up duration (in this study, originally 4.5 years (234 weeks), ranging from 7 months to almost 19 years): When limiting the follow-up duration to 156 or even 78 weeks, most changes disappeared. It is interesting to note that some tests even switched to an improvement over time, which could indicate practice effects.^34^ These improvements over time were most pronounced in the control group but were also observed in the biomarker groups for several outcome measures. Practice effects on outcome measures for episodic memory and attention observed over 78 weeks in controls were negated in most biomarker groups, which implies that the absence of improved performance over time is reflective of underlying AD pathology. This has previously been shown in cognitively unimpaired older adults.^35-37^ However, this contrast was not evident over 156 weeks, so it seems that practice effects and lack thereof might hold most utility over very short periods and disappear over slightly longer follow-up times before switching to decline. Meanwhile, the absence of decline over short follow-up durations is an important factor to be considered for trial design because relatively short trial durations are currently common practice in prodromal AD and mild AD dementia.^10,22-24,38^ Moreover, clinical trials are often stopped after futility analyses, which may show negative findings after limited follow-up even when changes may be observed over longer follow-up durations.^39,40^ A downside to longer trial duration is that it may increase participant burden or raise the barrier to participate, especially in preclinical stages where participants do not yet experience complaints and are less aware of their potential candidacy for trial participation. Hence, there is a need for more evidence on efficient trial design, weighing both the costs and benefits, to optimally inform potential participants.

This is an especially relevant issue when considering typical clinical trials enrolling cognitively normal participants known to be at risk of AD, which, in 2022, had an average duration of treatment of 159 weeks (i.e., just over 3 years).^25^ Even with follow-up duration of 156 weeks, many outcome measures investigated in this study did not seem to be responsive to change over time. With this study, we highlight that short study durations are unlikely to yield evidence of considerable cognitive decline using current cognitive and functional measures.

On the other hand, our findings corroborate previously demonstrated responsiveness to changes over time of various traditional pencil-and-paper cognitive outcomes in biomarker-defined groups.^31,41-44^ Several of these studies also report that more abnormal biomarkers are associated with steeper decline or decline on more outcomes. Thus, it seems that cognitive decline over time, in the context of AD pathology, can indeed be observed in those who are initially unimpaired, particularly when these individuals are followed for a considerable amount of time, that is, at least 4 or 5 years. A recently concluded solanezumab trial, although negative, was an example of a well-designed study, with relatively long follow-up time (240 weeks) and using the PACC as the primary outcome.^37^ In these asymptomatic, amyloid-positive individuals, a decline was found on this cognitive composite, which aligns with the findings from our amyloid-positive group. However, more than half of the amyloid-positive individuals in this trial were *APOE* ε4 carriers, and p-tau status was unknown. According to our results, in amyloid and p-tau–positive individuals, the use of PACC as the primary outcome is appropriate; however, in amyloid-positive individuals and amyloid-positive *APOE* ε4 carriers, a single neuropsychological test was more sensitive.

Yet, there are also findings that point to the contrary, with studies showing very limited or no decline in the cognitively unimpaired, especially when using psychometrically weak instruments and/or limited follow-up duration.^45-47^ Real-world effect sizes have been estimated to likely fall within the margin of error induced by heterogeneity of trial populations.^15^ Still, pencil-and-paper cognitive tests are ubiquitously used as primary and secondary outcomes. Although negative trial results have taught many important lessons,^14^ it seems that updating primary and secondary outcome measures is a lesson yet to be learned.

The above leads us to conclude that traditional pencil-and-paper tests may not be the best cognitive outcomes for clinical trials, at least not with current trial duration. Relying only on these outcomes that have been shown to be insensitive to change over short amounts of time puts the trial at a disadvantage. Without proper assessment tools, potential benefits from the intervention become more difficult, if not impossible, to detect. In recent years, promising new methods of assessment have been developed, including more appropriately challenging cognitive tasks and burst assessment. Based on the results we present in this study, we recommend cognitive and functional assessment be brought into the twenty-first century so that in the future, we are not left to wonder whether a treatment could have had a benefit had a more appropriate outcome measure been used.

Our study had a few limitations. First, these are retrospective analyses limited to the available data. Our average follow-up duration was 4 years, which is relatively short in a preclinical population.^48^ On the other hand, compared with trial duration, 4 years is quite long. While we investigated change over time on 2 composite measures, we could not re-create the measures exactly and substituted or omitted components of the measures we did not have access to. Second and related, these data were collected in a clinical cohort study in which neuropsychological assessments served both clinical and research purposes. Therefore, not all tests were administered to everyone, leading to inconsistent numbers of participants and observations per outcome measure. While this may complicate the comparison of results between outcome measures, we aimed to use as many assessments for each outcome measure as possible. Third, our sample was relatively young and highly educated, thus limiting the generalizability of our findings to older populations or individuals with fewer years of formal education. For both characteristics, it is known that they influence the rate of decline. According to cognitive reserve theory,^49^ highly educated individuals compensate for pathology for a longer period of time. Regarding age, while it is possible that cognitive decline in younger individuals may be less steep than in older individuals, it has also been shown that the disease follows a more aggressive trajectory in those with an earlier onset. A review showed that literature is inconclusive.^50^ Fourth, the plasma p-tau, *APOE* and amyloid-positive, and amyloid and CSF p-tau–positive biomarker groups had small sample sizes, which may have limited our power to detect change over time. Furthermore, the group of CSF p-tau–positive individuals was larger and younger than the group of amyloid and CSF p-tau–positive individuals. The amyloid cascade hypothesis holds that amyloid accumulates before p-tau,^1^ which would suggest that amyloid positivity should precede p-tau positivity. Important strengths of our study included the large sample of extensively phenotyped individuals because we had access to biomarkers, genetic information, and extensive cognitive testing. Another strength is our inclusion of a blood-based biomarker group because it constitutes a less invasive and more cost-efficient method for screening and monitoring disease progression. Future studies should replicate these findings as blood-based biomarkers and their cutoffs are further developed.

In conclusion, several neuropsychological, functional, and composite outcome measures detect changes over time in various biomarker-defined groups. When using these measures, AD prevention trials that aim to prove cognitive and/or functional benefits of their investigational drugs should use sufficiently long follow-up durations, exceeding the currently common duration of 3 years. Otherwise, well-validated digital and more sensitive cognitive tests are needed to optimally capture change over shorter time.

## Appendix Authors

**Table:**

Name	Location	Contribution
Mark A. Dubbelman, PhD	Alzheimer Center Amsterdam, Neurology and Amsterdam Neuroscience, Neurodegeneration, Amsterdam UMC, Vrije Universiteit Amsterdam, the Netherlands	Drafting/revision of the manuscript for content, including medical writing for content; study concept or design; analysis or interpretation of data
Heleen M.A. Hendriksen, MD	Alzheimer Center Amsterdam, Neurology and Amsterdam Neuroscience, Neurodegeneration, Amsterdam UMC, Vrije Universiteit Amsterdam, the Netherlands	Drafting/revision of the manuscript for content, including medical writing for content; analysis or interpretation of data
John E. Harrison, PhD	Alzheimer Center Amsterdam, Neurology and Amsterdam Neuroscience, Neurodegeneration, Amsterdam UMC, Vrije Universiteit Amsterdam, the Netherlands	Drafting/revision of the manuscript for content, including medical writing for content; study concept or design; analysis or interpretation of data
Everard G.B. Vijverberg, MD, PhD	Alzheimer Center Amsterdam, Neurology and Amsterdam Neuroscience, Neurodegeneration, Amsterdam UMC, Vrije Universiteit Amsterdam, the Netherlands	Drafting/revision of the manuscript for content, including medical writing for content
Niels D. Prins, MD	Brain Research Center, Amsterdam, the Netherlands	Drafting/revision of the manuscript for content, including medical writing for content
Lior A. Kroeze, MS	Alzheimer Center Amsterdam, Neurology and Amsterdam Neuroscience, Neurodegeneration, Amsterdam UMC, Vrije Universiteit Amsterdam, the Netherlands	Drafting/revision of the manuscript for content, including medical writing for content; major role in the acquisition of data
Lois Ottenhoff, MS	Alzheimer Center Amsterdam, Neurology and Amsterdam Neuroscience, Neurodegeneration, Amsterdam UMC, Vrije Universiteit Amsterdam; Brain Research Center, Amsterdam, the Netherlands	Drafting/revision of the manuscript for content, including medical writing for content
Mardou M.S.S.A. Van Leeuwenstijn	Alzheimer Center Amsterdam, Neurology and Amsterdam Neuroscience, Neurodegeneration, Amsterdam UMC, Vrije Universiteit Amsterdam, the Netherlands	Drafting/revision of the manuscript for content, including medical writing for content; major role in the acquisition of data
Inge M.W. Verberk, PhD	Alzheimer Center Amsterdam, Neurology; Amsterdam Neuroscience, Neurodegeneration; and Neurochemistry Laboratory, Department of Laboratory Medicine, Amsterdam Neuroscience, Amsterdam UMC, Vrije Universiteit Amsterdam, the Netherlands	Drafting/revision of the manuscript for content, including medical writing for content
Charlotte E. Teunissen, PhD	Alzheimer Center Amsterdam, Neurology; Amsterdam Neuroscience, Neurodegeneration; and Neurochemistry Laboratory, Department of Laboratory Medicine, Amsterdam Neuroscience, Amsterdam UMC, Vrije Universiteit Amsterdam, the Netherlands	Drafting/revision of the manuscript for content, including medical writing for content
Elsmarieke M. van de Giessen, MD, PhD	Department of Radiology & Nuclear Medicine and Amsterdam Neuroscience, Neurodegeneration, Amsterdam UMC, Vrije Universiteit Amsterdam, the Netherlands	Drafting/revision of the manuscript for content, including medical writing for content
Argonde C. Van Harten, MD, PhD	Alzheimer Center Amsterdam, Neurology and Amsterdam Neuroscience, Neurodegeneration, Amsterdam UMC, Vrije Universiteit Amsterdam, the Netherlands	Drafting/revision of the manuscript for content, including medical writing for content; major role in the acquisition of data
Wiesje M. Van Der Flier, PhD	Alzheimer Center Amsterdam, Neurology; Amsterdam Neuroscience, Neurodegeneration; and Department of Epidemiology & Data Science, Amsterdam UMC, Vrije Universiteit Amsterdam, the Netherlands	Drafting/revision of the manuscript for content, including medical writing for content; major role in the acquisition of data; study concept or design; analysis or interpretation of data
Sietske A.M. Sikkes, PhD	Alzheimer Center Amsterdam, Neurology and Amsterdam Neuroscience, Neurodegeneration, Amsterdam UMC, Vrije Universiteit Amsterdam, and Department of Clinical, Neuro and Developmental Psychology, Faculty of Behavioral and Movement Sciences, Vrije Universiteit, Amsterdam, the Netherlands	Drafting/revision of the manuscript for content, including medical writing for content; major role in the acquisition of data; study concept or design; analysis or interpretation of data

## Glossary

AD: Alzheimer disease
ADC: Amsterdam Dementia Cohort
A-IADL-Q: Amsterdam Instrumental Activities of Daily Living Questionnaire
AVLT: Auditory Verbal Learning Test
BNT: Boston Naming Test
CFC: Cognitive-Functional Composite
DS: digit span
GDS: Geriatric Depression Scale
LDST: Letter Digit Substitution Test
LMM: linear mixed model
MMSE: Mini-Mental State Examination
PACC5: Preclinical Alzheimer's Cognitive Composite
p-tau: phosphorylated tau
RBMT: Rivermead Behavioral Memory Test
SCWT: Stroop Color-Word Test
TMT: Trail Making Test
